# Using Single Colors and Color Pairs to Communicate Basic Tastes

**DOI:** 10.1177/2041669516658817

**Published:** 2016-07-07

**Authors:** Andy T. Woods, Charles Spence

**Affiliations:** Crossmodal Research Laboratory, Oxford University, UK

**Keywords:** color, basic tastes, crossmodal correspondences, synesthesia

## Abstract

Recently, it has been demonstrated that people associate each of the basic tastes (e.g., sweet, sour, bitter, and salty) with specific colors (e.g., red, green, black, and white). In the present study, we investigated whether pairs of colors (both associated with a particular taste or taste word) would give rise to stronger associations relative to pairs of colors that were associated with different tastes. We replicate the findings of previous studies highlighting the existence of a robust crossmodal correspondence between individual colors and basic tastes. However, while there was evidence that pairs of colors could indeed communicate taste information more consistently than single colors, our participants took more than twice as long to match the color pairs with tastes than the single colors. Possible reasons for these results are discussed.

## Introduction

Spence et al. (2015) recently summarized the literature documenting the existence of robust crossmodal correspondences between colors (or color words) and basic tastes or taste words (Spence, 2011, 2012a). They reported that across a number of studies conducted over the last 30 years or so, people have been shown to reliably associate certain colors with specific basic tastes (see Table 1, for a summary).

**Table 1.** Summary Table Showing the Two Colors That Previous Research Suggests Are Most Strongly Associated With Each of the Four Basic Tastes.

Spence et al. (2015)		Favre and November (1979)	
Taste word	Colors	Taste word	Colors
Sweet	Red, pink	Sweet/Sweetish	Orange-yellow to red/pink
Sour	Green, yellow	Acid	Yellowish green to greenish yellow
Salty	White, blue	Salted	Grey with pale green or with pale blue
Bitter	Black, purple	Bitter	Navy blue, brown, olive-green, violet

*Note.* Based on the summary provided by Spence et al. (2015) and the color-taste recommendations for designers specified in Favre and November (1979).

Now, while the matching of tastes (or taste words) to individual color patches is reasonably consistent (i.e., it is significantly nonrandom; see Spence et al., 2015, for a summary of the literature; see Razumiejczyk, Macbeth, Marmolejo-Ramos, & Noguchi, 2015, for a recent demonstration that taste words can simulate actual taste experiences), the question to be addressed in the present study was whether presenting pairs of colors (either congruent—such as red and pink for sweet or incongruent—such as red and green for sweet; see Table 1) would lead to even more consistent taste matching (cf. Jacquot, Velasco, Spence, & Maric, 2016; Schifferstein & Howell, 2015, for research on the analogous question of whether fragrance or aroma is better matched to palates of colors vs. single color patches).

The participants in the present study were shown a random sequence of eight colors, either presented in isolation or else in one of a set of 16 color pairs. As well as broadly replicating the pattern of results that has been documented across a number of previous studies (see Spence et al., 2015, for a review), there were a number of novel predictions arising from the present research:

Hypothesis 1: Congruent color pairs would give rise to more consistent matching to taste than a single color patch.

Hypothesis 2: Congruent color pairs would be responded to more rapidly that incongruent color pairs and people would be more confident about their responses.

We were also interested in investigating whether participants’ responses to incongruent color pairs would be driven by the individual color with the strongest match to a particular taste, or perhaps to the most subjectively intense color, or perhaps by something else.

Previously, designers and marketers have, on occasion, made suggestions concerning the optimal combination of colors with which to convey a particular taste (e.g., see Favre & November, 1979, p. 79; the suggestions of which are outlined in Table 1). It is, however, typically unclear on what basis such recommendations have been made. Hence it is difficult to know, in hindsight, how much weight should be put on such suggestions. It is also unclear whether such crossmodal associations may have changed over the years (just think, e.g., of how transparent blue drinks have, over the last decade or so, come to be associated with raspberry-flavor; see Spence, 2015a). By contrast, here we provide a scalable Internet-based testing approach for assessing the strength of the association between two stimuli (here taste words and colors).

## Methods

### Participants

Two hundred and one participants (94 female) were recruited from Amazon’s Mechanical Turk to take part in the study in return for a payment of 1.50 US dollars. The participants ranged in age from 18 to 70 years (*M* = 35.0 years, *SD* = 11.58). The experiment was conducted on July 1, 2014, from 2:00 p.m. GMT onwards, over a period of 1-hour (see Woods, Velasco, Levitan, Wan, & Spence, 2015, for a recent methodological overview of Internet-based psychological research). The participants took an average of 368 s (*SD* = 185) to complete the study. All of the participants provided their informed consent prior to taking part. The experiment was reviewed and approved by the Central University Research Ethics Committee at Oxford University and was carried out in accordance with the World Medical Association Helsinki Declaration as revised in October 2008 (http://www.wma.net/en/30publications/10policies/b3/index.html).

### Stimuli

Eight color squares (75 pixels × 75 pixels) were used as stimuli. They were either presented individually or else were combined into one of 16 pairs of stimuli. The colors presented individually were green, yellow, red, pink, blue, white, black, and purple, a subset of the so-called *Internet safe* colors (hex codes were 00FF00, FFFF00, FF0000, FFC0CB, 0000FF, FFFFFF, 000000, and 800080, respectively). The color pairs comprised two of these color patches: redPink*, blueWhite*, yellowGreen*, blackPurple*, redBlue, redYellow, redPurple, whiteYellow, whitePurple, whitePink, greenPurple, greenBlue, greenPink, blackPink, blackYellow, and blackBlue (* suffixed patches were thought likely to be congruent, see Table 1; The remaining colors in each pair were randomly assigned under the proviso that each primary color was presented exactly four times across all of the stimulus pairs). The respective patches in each stimulus pair were always separated from each other by 5 pixels. The stimulus pairs were either stacked vertically, one on top of the other, or else were arranged horizontally. Whether a participant saw the color pairs arranged vertically or horizontally was determined randomly at the start of each participant’s experimental session. The vertical or horizontal position of the color patches in each pair was determined randomly at the start of each trial. The background against which the stimuli were presented was gray (hex code 7A7A79).

### Apparatus

Given that the experiment was conducted online, the apparatus varied by participant. The experiment utilized “full screen” mode (i.e., utilizing the entirety of the participant’s monitor) and took place within a 1024 × 768-pixel box in the centre of the screen, irrespective of the size of the participant’s monitor. The experiment was conducted on the Internet using the Adobe Flash-based version of Xperiment (http://www.xperiment.mobi).

### Design

A within-participants experimental design was used with all of the participants undertaking all 24 of the experimental trials (8 single colors and 16 color pairs), in a random order. There were four dependent variables: the taste-word chosen for each color stimulus, the time taken to decide on that taste-word, the participant’s confidence in their rating, and lastly, how subjectively intense a given single color stimulus was rated as being.

### Procedure

**Figure 1.**
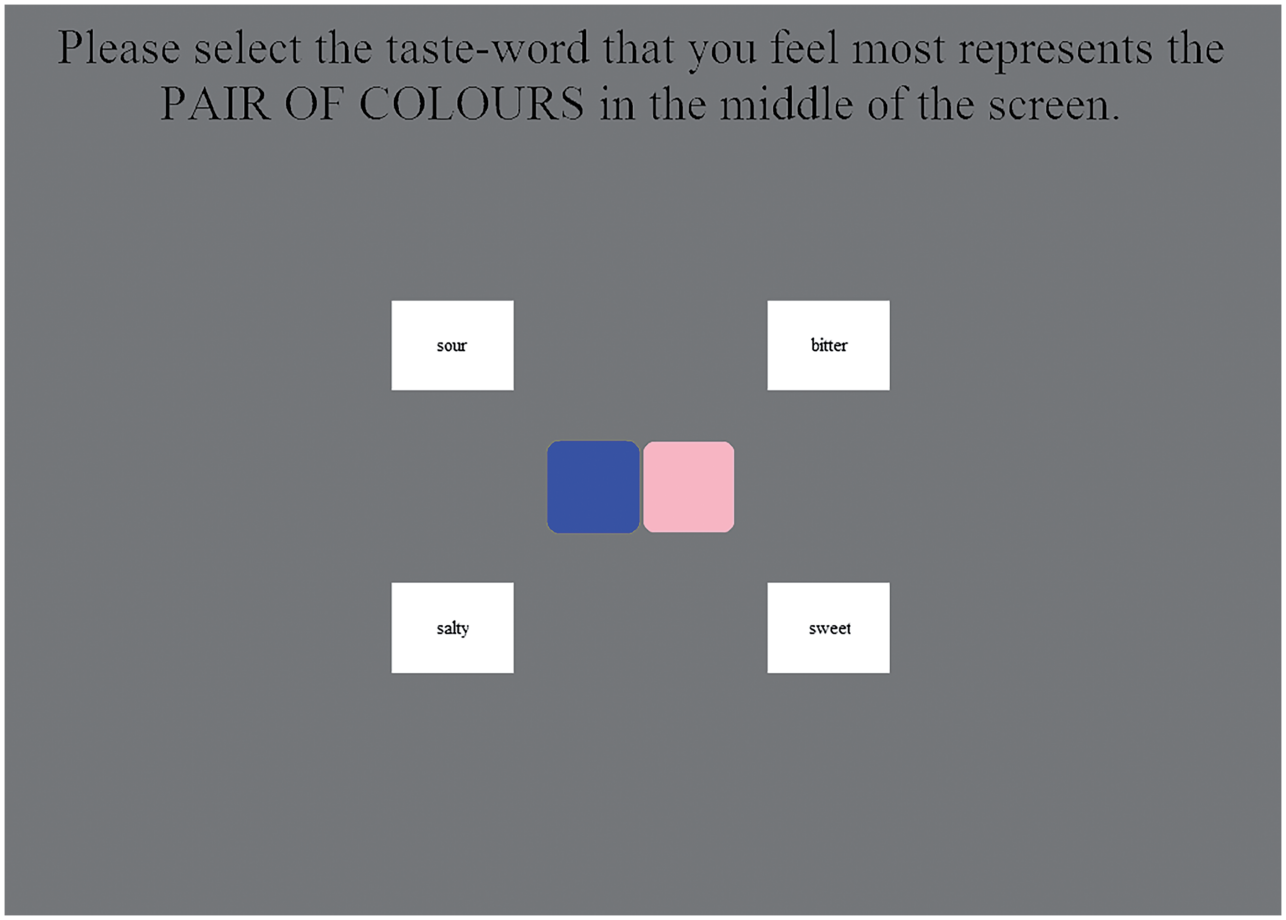
Screenshot illustrating the first part of the experimental task. The participants had to judge which of the four taste words best matched the pair of color patches shown in the center of the screen.

The participants had to select the taste-word that they felt best represented the color stimulus (the position of the response words, see Figure 1, was randomized across participants). After making this decision, the participant was asked “Please rate how confident you are in your decision.. They were instructed to respond on a 5-point scale (labelled *not at all confident*, *not confident*, *undecided*, *confident*, and *very confident*). The participants were then asked “Please rate how much you liked the pair of colors” (*dislike very much*, *dislike*, *neither*, *like*, or *like very much*). In those trials in which a single color patch was presented, the participant was also (finally) asked “Please rate how intense you found the color” (*not at all intense*, *a little intense*, *somewhat intense*, *intense*, or *very intense*; However, at the suggestion of a reviewer, we decided not to report analyses on intensity due to potential ambiguity for participants as to the meaning of subjective intensity).

At the end of the session, participants were presented with the eight color patches used in the main study and eight colors words next to each patch. The participants had to try and select the correct color word for each color patch. Seven of the participants failed to complete the catch trial correctly. Their data were not, however, excluded from the subsequent data analyses due to their low number, and because they only failed the task for two or fewer colors—thus, perhaps, suggesting an accidental error rather than anything else.

## Results

We start by analyzing the data from the eight single color trials, before analyzing the data from the color pairs.

### Single Colors

**Figure 2.**
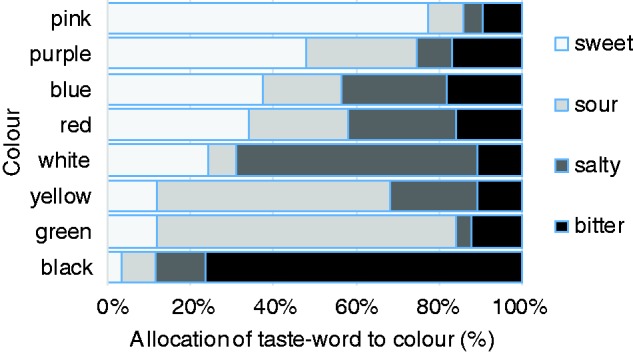
Proportion of trials on which each taste word was associated with each of the eight individually presented color patches. Broadly consistent with the predictions emerging from previous research (see Spence et al., 2015, for a summary), the color that was most frequently associated with sweet was pink, green with sour (though yellow was not too far behind), white was associated with salty, and black with bitter.

**Figure 3.**
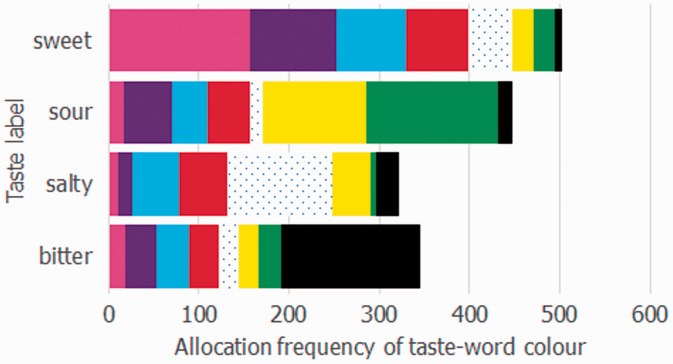
The frequency with which a particular color was assigned to a given taste word. Once again, this figure makes clear that the strongest color-taste word associations were for pink (and to a lesser extent purple) and sweet, green (and to a lesser extent yellow) and sour, white and salty, and black with bitter. What is also clear from visual inspection of this figure is that the red, blue, and possibly also purple color patches were reasonably evenly distributed across each of the four possible taste words. This means that these colors (e.g., when presented as part of a color pair) should presumably not have biased people toward any one specific taste word.

The frequency with which the participants assigned each of the eight color patches to one of the taste words is shown in Figure 2. Bonferroni corrected chi-square tests revealed that all of the color patches were assigned to tastes with a frequency that differed significantly from that expected by chance (13.80 < χ^2^(3) < 294.75, red *p* < .05, blue *p* < .01, *p* < .001, for the remainder; n.b. the range of chi-square values that all color patches were banded within is reported). To give an overall impression of the colors that the participants judged most representative of a given taste word, the data from Figure 2 are replotted on a taste-by-taste basis in Figure 3. Note, however, that no statistics were performed on this representation of the data, as the taste allocations per color depended on how a given taste had been allocated to the other colors. Note also that the dominant colors for sweet (pink, purple*), sour (green, yellow), salty (white, blue), and bitter (black, blue*) shown in Figure 3 do not tally with those reported in Table 1 (colors * suffixed were the colors red and purple, respectively, in the table). Here it is worth remembering that the nature of our task meant that if a given color was strongly associated with more than one taste (such as red, see Figure 2), its scores for a given taste (e.g., sweet) would be much reduced as compared with a color with fewer associations overall (e.g., purple). Adopting a forced choice task, in which the participant must decide which single *color* out of several best matches a given taste (as opposed to which *taste* befits a given color) would not be expected to be so affected, and it is in such tasks, as reported by Spence et al. (2015) that the data in the table were derived.

**Figure 4.**
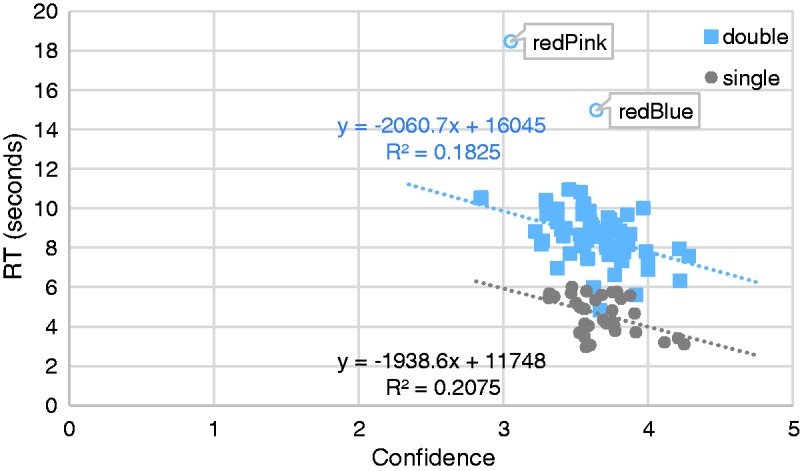
The relationship between the time taken to decide which taste best befits a given stimulus (single color or color pair) and the participant’s confidence in their rating. Unfilled data points were suspected of being outliers and so were excluded from the data analyses. The significant correlations show that the more confident the participants were in their allocation of a color patch to a taste word (i.e., the higher the value on the X-axis), the more rapidly they responded, as might have been expected.

The average reaction times (RTs), confidence ratings, and frequency of allocation were computed for each color-taste mapping (n.b., RT outliers, defined as falling outside a belt around of the mean of ±3 standard deviations, were replaced with either the mean +3 standard deviations or the mean −3 standard deviations, whichever was closer, Field, 2009). As might have been expected, RT and Confidence were negatively correlated, *r* = −.46, *p* < .01, *n* = 32, 95% CI [−.13, −.71] (Pearson correlation coefficient reported here and henceforth; correlation CIs here and henceforth were calculated by means of a 10,000 sample bootstrap). Specifically, the more confident the participants were in their allocation of a color patch to a taste word, the quicker they made their response (see Figure 4). No other significant correlations were observed.

Taken together, the results of the allocation of the individual color patches to taste words clearly show that black is most commonly associated with bitter; White with salty; Green, closely followed by yellow with a sour taste; and pink (followed by purple) was primarily associated with sweetness. Having established a basic mapping between individual color patches and basic tastes (or taste words), we now analyzed the data from the color pairs. Note that the critical question here is whether the addition of a second color to these dominant color-taste associations established on the basis of the single color trials would change participants’ performance in a meaningful manner. To put this notion in context, one might legitimately consider whether participants would find it easier to associate the green and yellow color pair with sourness than if they had to associate either of these colors individually with a sour taste. By contrast, given the results shown in Figure 3, one would not really expect the addition of a red, blue, or to a lesser extent purple color patch to exert much of an influence on participants’ responses to the color pairs, since the above analyses revealed that these three colors were roughly equivalently associated with each of the four basic tastes (and hence presumably did not carry much information).

### Two Colors

**Figure 5.**
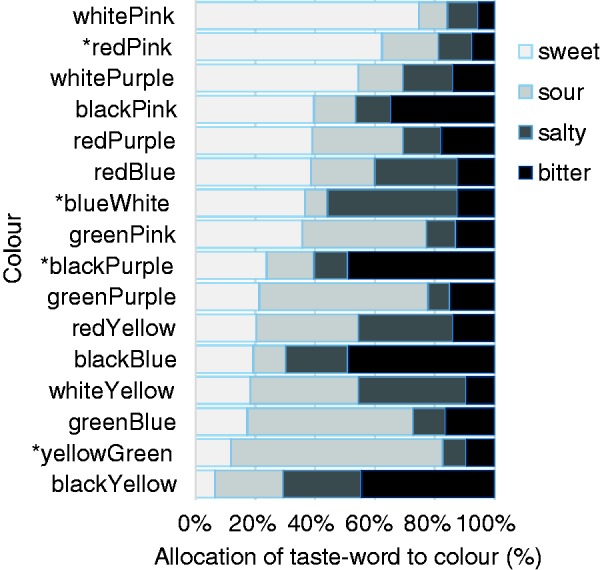
Proportion of trials in which each taste word was associated with each of the 16 color pairs (congruent pairs are prefixed with an *). As can be seen from visual inspection of this figure, the sweet-pink, the yellow- and green-sour, white-salty, and black-bitter correspondences stand out. Intriguingly, though, when white (individually salty) and pink (individually sweet) were paired, it was the sweet association that dominated participants’ responses. The highest percentage of sour associations was with the yellow-green color pair, as might have been expected given the results reported earlier (see Figure 3).

**Figure 6.**
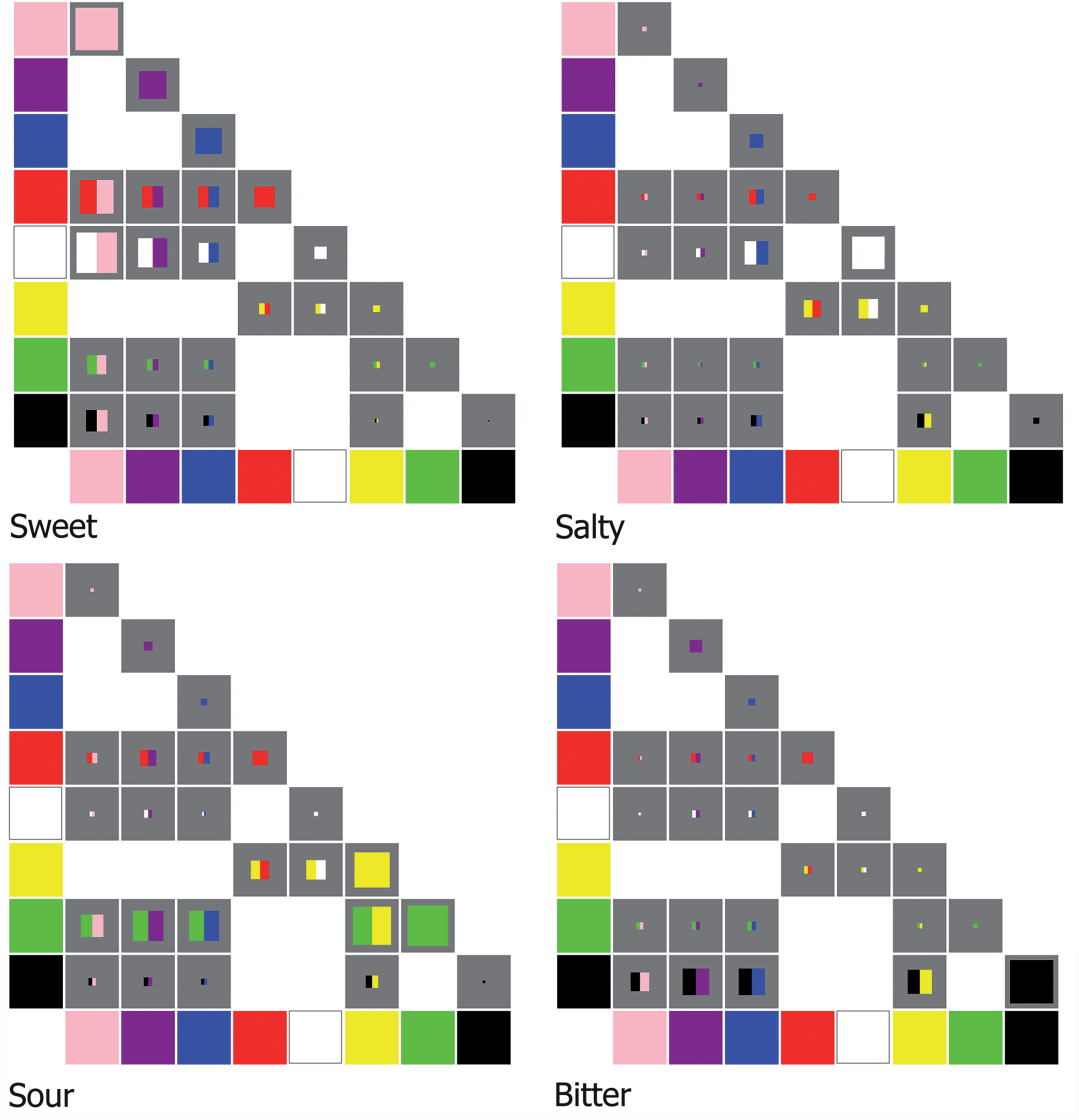
Four color matrix plots depicting the frequency with which the different colors and color-pairs were selected for each of the four basic taste terms used in the present study. For each plot, the eight colors are shown along the x- and y-axes. At the intersection of the color on each axis, the presence of a gray box indicates that a specific combination of colors was tested for in the experiment (no gray box indicating that this color-pair was not tested). The square color patch within each gray box consists of the color, or colors, that was or were presented to participants in order to assign a basic taste to. The size of the color patch, relative to the size of the gray box, represents the number of participants who chose that color patch to reflect the given basic taste (the length of the patch as compared with the length of the gray box representing the ratio of participants choosing that particular taste for the given color). So, if the patch were to fill the entire gray box, this would imply that *every* participant selected the given color for the particular matrix’s taste; if the gray box were, however, to be empty, this would imply that *none* of the participants selected that color.

Bonferroni corrected chi-square tests revealed that tastes were assigned to each color with a frequency that was significantly different than that expected by chance (22.20 < χ^2^(3) < 267.78, *p* < .001; see Figure 5; the data for single and pair colors have been plotted in Figure 6 to aid comparison).

The average RTs, confidence ratings, and frequency of allocation were computed for each color-taste mapping. As for the one-color analyses reported earlier, RT and confidence correlated negatively, *r* = −.45, *p* < .001, *n* = 64, 95% CI [−.21, −.61]. Once again, the more confident the participants in their allocation of the color pairs to the individual taste words, the more quickly they responded (see Figure 4). The frequency or “strength” of association correlated positively with confidence *r* = .44, *p* < .001, *n* = 64, 95% CI [.12, .65].

Interestingly, however, there was no evidence for our second hypothesis, namely that any of the color pairs (mean 8520 ms, 95% CI [8209 ms, 8830 ms]) would be associated with a given taste more rapidly than was the congruent single color (4607 ms, 95% CI [4256 ms, 4957 ms]), *t*(92) = 15.71, *p* < .001 (independent samples). Indeed, it took our participants almost twice as long to allocate color pairs to a particular taste than it did to allocate single colors. This is clearly visible in Figure 4.

**Figure 7.**
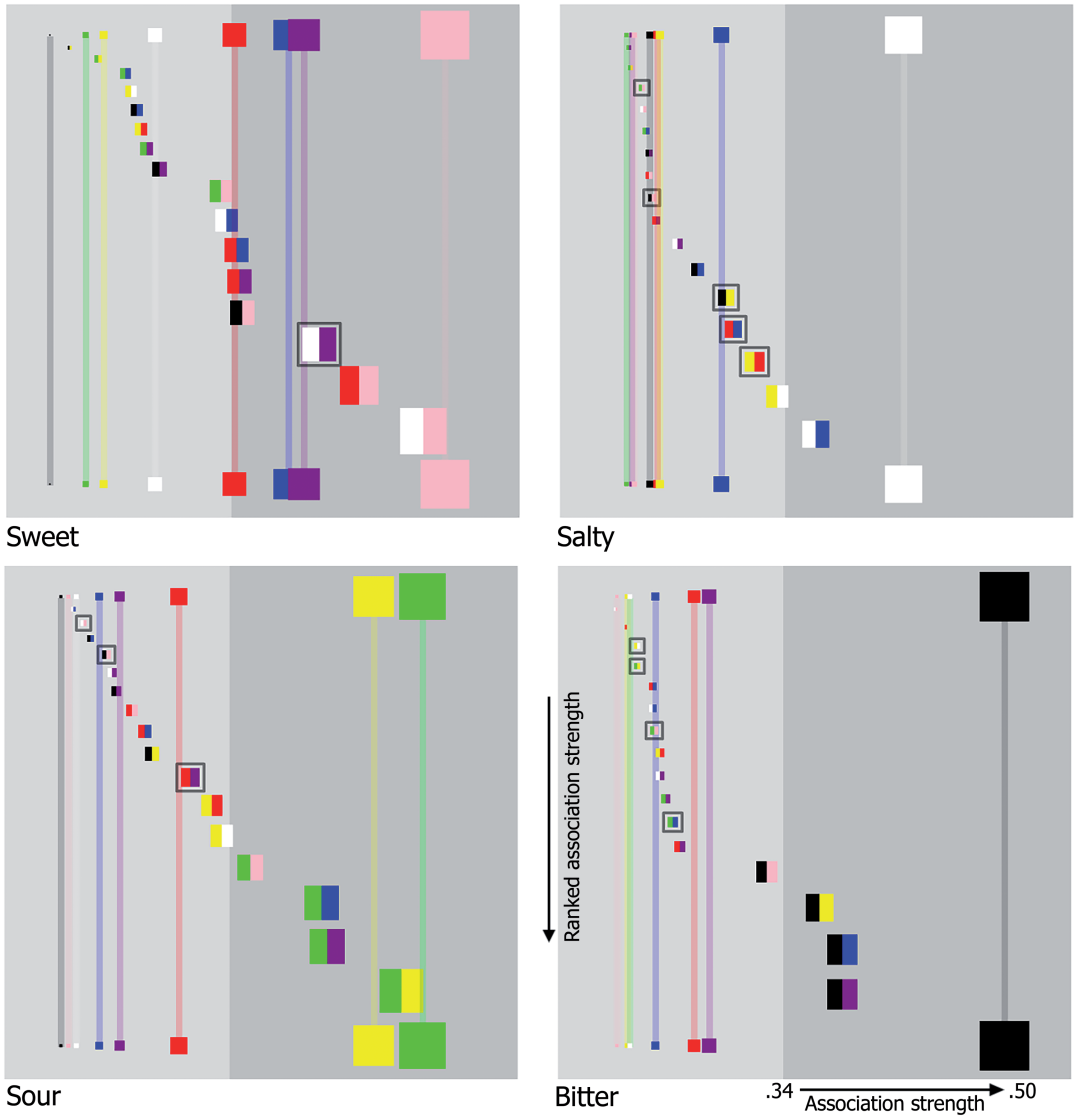
Four color matrix plots as specified in Figure 6. Now, the horizontal position of each patch represents the strength of association (as well as the size of each patch) with rightward-positioned patches being more strongly associated than those on the left-hand side (the more participants selecting a given color for a given taste, the stronger the association; the horizontal axis being expressed as a ratio in terms of this value over the total number of participants). Single color patches were presented horizontally along the top and bottom of each plot. In terms of their vertical placement, double color patches have been arranged from the weakest association (top) to the strongest (bottom) in each plot. For illustrative purposes, double color patches that were more strongly associated with a given taste than each of their constituent colors have been encapsulated in a gray highlighting box. The background of each plot was colored light gray and dark gray, the latter signifying the region in which a particular taste was selected for patches by participants at a level that was significantly higher than expected by chance (*p* < .001; the experiment was simulated 100,000 times and the maximum number of consistent “by-chance” selections over all colors × tastes per virtual-experiment was used to determine this).

Figure 7 demonstrates that of all of the colors pairs, only the whitePurple pair was more strongly associated with a given taste (salty) than were the constituent colors when presented individually. Here, though, it should be noted that all of the colors were displayed against a gray background.

### Further Analyses

Up until this point, we have considered the data at a group level (across participants), in terms of whether or not a given color was associated with a particular taste or whether two colors are congruent in that they associate with the same taste. However, it is important to note that congruency can also be considered on an individual basis. To give a concrete example, just take the pairing of yellow and green with the sour taste. It is possible that our participants either paired green with sour or yellow, but not with both of the colors. Should this have been the case, it might not have been so surprising to find that the combined yellow-green color pair was not assigned to sour more rapidly than either of the individual color patches, since for no participant (in this hypothetical case) were the two colors both associated with the same taste sour. The upshot of all this is that the group-level analysis may be less sensitive compared with one conducted on an individual-by-individual basis. We adopt the latter approach in the subsequent analyses.

If our first hypothesis were to be correct, namely that congruent color pairs would give rise to more consistent taste pairings than those for single colors, then one would expect that color pairs whose individual colors in the single color task were assigned as the same taste would also be more likely to be classified as that taste than otherwise. This was indeed the case. Specifically, out of the 733 congruent single color or taste assignments, color pairs (count 482, see Table 1) were 1.92 times more likely to share the same taste than not (251), χ^2^(1) = 72.805, *p* < .001. There was, however, no evidence for the second hypothesis; specifically, that the congruent color pairs would be responded to more rapidly that incongruent color pairs (and more confidently assigned): As shown in Table 2, the confidence intervals for confidence overlapped, as did those for RT.

**Table 2.** Twenty percentage of trimmed mean of confidence scores and RT, and their 95% confidence intervals (calculated via the trimpb function in the WRS R package, Wilcox & Schoenbrodt, 2009), for the four groups of data (as defined in the table).

	Percent (frequency)	Mean confidence (95% CI)	Mean RT (95% CI)
Single color patches associated with different tastes, and double patch associated with same taste as either one of the single color patches	58.30% (1885)	3.56 (3.50, 3.62)	7836 (7651, 8020)
Single and double patches all associated with different tastes	19.00% (614)	3.47 (3.38, 3.55)	7947 (7627, 8267)
Single and double patches all associated with the same taste	14.91% (482)	3.67 (3.59, 3.75)	7331 (6992, 7670)
Both color patches individually associated with same taste, but color pair association is different	7.77% (251)	3.51 (3.36, 3.65)	7219 (6644, 7795)

Despite previously correcting for outliers, visual inspection of a plot of confidence rating against RT suggested that many RT data points were still outlying. Robust statistics are thus reported.

A similar analysis was suggested by a reviewer. If the colors in a color pair both strongly (weakly) associate with a given taste, one would predict a strong (weak) relationship between that pair and the given taste. If, though, one color was strongly related but the other was less so, the strength of the color-pair to taste relation would presumably be expected to be diluted somewhat. We therefore developed a new measure of association strength for each single color to taste association, by dividing the number of participants deciding on a given taste for the particular color, by the strongest color to taste association for a particular taste (e.g., 156 participants regarded Pink as sweet as compared with 17 individuals for Pink as Sour; thus the score for Pink Sweet was 156/156 = 1, while the score for Pink Sour was 17/156 = .12). For each color-pair, we multiplied or added the individual association strength measures for each color. We found that these permuted scores were strongly associated with the actual scores from the color pair data both when multiplied, *r* = .84, *p* < .001, *n* = 64, 95% CI [.77, .90], and when combined additively, *r* = .92, *p* < .001, *n* = 64, 95% CI [.87, .96], thus providing empirical support for our first hypothesis (consistent with the conclusions of the immediately preceding analysis). Although confidence intervals marginally overlapped, be it only descriptively, the multiplied strengths of the color pairs were a better predictor of the dual color associations than their strongest individual color association, *r* = .79, *p* < .001, *n* = 64, 95% CI [.67, 89].

## Discussion

Analysis of the results of the present study confirmed previous findings highlighting the existence of robust crossmodal correspondences between individual colors and basic taste (words). Furthermore, there was also some evidence in support of our first hypothesis but no real evidence for the second, concerning how participants would respond to the color pairs that were set out in the Introduction section. In terms of our first hypothesis, where the prediction was that congruent color pairs would give rise to a more consistent matching to taste than individual color patches, with our original analysis only the whitePurple patch was assigned more strongly to sweet than either of its constituent colors when presented individually (i.e., at a level that was greater than that expected by chance). This was not the case for any of the other 15 color pairs. The results of a follow-up analysis (the last in the “Results” section) suggested by a reviewer supported our first hypothesis, with the combined strengths of association from the single color task strongly predicting how likely a given color pair would be assigned to a given taste (more so, indeed, than predicted from only the strongest single color). In terms of our second hypothesis, though, there was no support for the suggestion that participants would respond more rapidly to congruent color pairs than to single colors. Instead, the participants actually took *much* longer (in fact, almost twice as long) to pair tastes with color pairs as compared with single colors. This could imply some internal debate as to which color the participant decided to pair up with a taste, assuming that they experienced some conflict on this score.^1^ As pointed out by a reviewer, this could also mean that the colors are individually assessed in a serial manner and then assimilated, which might well take some time (though see the literature on the redundant signals or target effect, mentioned later). In light of this, the partial support for Hypothesis 1 could also be the result of a quite different set of (higher order) processes (e.g., operating at a decisional level) than would typically occur in everyday perceptual experience. More research is thus needed in order to check ecological validity and to rule out the earlier alternative explanations for this finding.

Of course, when considering the use of color pairs, one needs to think not just about whether a particular pair of colors effectively conveys a specific taste but also about how pleasant people rate the particular combination of colors (see Palmer, Schloss, & Sammartino, 2013). Here, the literature on complementary colors is certainly relevant. In one recent study, Schloss and Palmer (2011) distinguished amongst three types of judgments for figure-ground color pairs: (a) pair preference (how much the two colors are liked together), (b) pair harmony (how well the colors go together, regardless of preference), and (c) figural preference (how much the foreground color is liked when viewed against a colored background). However, given that color pairs were generally less strongly associated with a basic taste than were the individual color patches, such concerns about the pleasantness of color combinations become moot in the present case.

Several other interesting findings, of both basic and applied relevance, emerged from the present study. On the more theoretical side, our results add weight to recent findings suggesting that different crossmodal correspondences do not necessarily sum in an additive manner. This is most clearly demonstrated for the yellow-green color pair, as seen in Figure 7, for the sour taste. In a previous study, Woods, Spence, Butcher, and Deroy (2013) found that augmenting the Bouba/Kiki task with happy and sad faces had no real effect of how people assigned round and angular shapes to the “bouba” and “kiki” labels (note here that facial emotion was hypothesized to be related on the same underlying dimension as the bouba/kiki sounds and thus could have been expected to influence participants’ classification performance).

One should consider, though, what exactly it is about a given color that leads to it being assigned as a given taste. One possibility here is that it is actually some specific or general food memory prompted by the color, or indeed even hue, that gives rise to a particular assignment to taste (as suggested in the discussion of Woods, Poliakoff, Lloyd, Dijksterhuis, & Thomas, 2010), rather than the color itself. For example, the color pink may bring candyfloss to mind, and it is this association rather than the color itself that is thought of as sweet (though note that not all authors believe that correspondences are necessarily grounded in specific experiences of environmental objects that possess both features). Of relevance here is the fact that two colors presented side-by-side most likely provoke quite different memories; even if both individually would be thought of as sweet, would they combine in an additive fashion^2^ as hypothesized here? If the combination is not actually experienced together in real life, then the answer is probably no.

These results should also be of interest to the fields of marketing and design (e.g., to packaging designers wishing to more effectively convey the taste of a product; note there is not complete agreement between the color-taste matches we experimentally determined here and those detailed in design books such as Favre & November, 1979, see Table 1). Our results certainly support the suggestion that distinctive individual colors would appear to convey taste information more effectively than combinations (specifically pairs) of colors. Of course, it is true that few products are actually sold on the basis of their taste alone (in the gustatory sense of the term, excepting of course sugar and salt), but rather on the basis of their flavor. The hope is that the method outlined here could be used in the future to address the crossmodal correspondences between color and flavor. Thinking more generally, the research outlined here can be fit within the field of synesthetic design (see Belli, 2001; Haverkamp, 2014; Spence, 2012b, 2013, 2015b; Velasco, Salgado-Montejo, Marmolejo-Ramos, & Spence, 2014; Wan, Woods, van den Bosch, McKenzie, Velasco, & Spence, 2014). One could also consider how this approach might, in the future, be extended to investigate the meaning of, for example, pill color (see Wan, Woods, Velasco, Salgado-Montejo, & Spence, 2015).

The results reported here, then, do not support the notion that pairs of colors can do a better job at communicating a particular taste (word), at lead not in terms of generating a faster, more intuitive response. A separate, but in some ways related question that we hope to address next concerns whether the incorporation of shape (or curvilinearity) to the designs can be used to further improve performance (improve in the sense of resulting in even performance that is even more consistent). The logic here being that people also show robust associations between tastes (or taste words) and shapes (see Spence & Deroy, 2013; Velasco, Woods, Deroy, & Spence, 2015, for a review).

## Notes

1 We are currently exploring whether more subtly paired-up colors (which we hope would remove the need for such internal debate), with one in the foreground, the other in the background would lead to different color-taste pairs.

2 From the perspective of object recognition, the fact that the taste properties of different food memories do not combine makes sense. For example (be it perhaps a little tangential), the Redundant Signals or Target Effect (CS1; see Engmann & Cousineau, 2013, for an overview) leads to faster detection when there are multiple redundant cues (processed in parallel) demanding the same response as compared with when there is just one. Indeed, some would argue that one of the critical processes in multisensory perception is to ensure that *unrelated* sensory cues are not erroneously combined (Spence, 2010).
